# The concept of exposure when selecting comparison groups for determining individual susceptibility to addiction to cigarette smoking

**DOI:** 10.1371/journal.pone.0214946

**Published:** 2019-04-11

**Authors:** Indiara W. Henn, Luciana R. A. Alanis, Adriana Modesto, Alexandre R. Vieira

**Affiliations:** 1 Graduate Program in Dentistry, Pontifícia Universidade Católica do Paraná, Curitiba, PR, Brazil; 2 Departments of Oral Biology and Pediatric Dentistry, School of Dental Medicine, University of Pittsburgh, Pittsburgh, PA, United States of America; Ohio State University, UNITED STATES

## Abstract

Smoking is a leading cause of preventable death. The effect of tobacco is even more contundent in people with mental illness and, in general, cigarette smoking addiction is influenced by genetic factors. The opioid system is involved in the mesolimbic reward system, which is of great importance in addictive behaviors, such as smoking and is influenced by genes such as the *OPRM1*. The aim of this study was to evaluate if selecting a comparison group that include light smokers versus people that never smoked impacts the results of genetic association studies. In addition, to evaluate the genetic association in different groups of smokers by analyzing independent covariates such as mental illness and clinical dental data. All subjects were participants of the Dental Registry and DNA Repository project. Genotyping was carried out using TaqMan chemistry for two markers in *OPRM1* (rs553202 and rs7755635). Logistic regression analyses were performed as implemented in PLINK. The established value for alpha was 5%, and the Hardy-Weinberg equilibrium was evaluated by the chi-square test with one degree of freedom for each marker. 1,897 patients were included, which were allocated to eight distinct groups, according to the frequency and quantity of cigarettes smoked and mental illness status. There was no significant association between the two markers in *OPRM1* and smoking. When mental illness and dental clinical data (tooth loss, dental caries, and periodontitis) were used as covariates, there were associations between heavy smoking and *OPRM1*, when non-smokers were used as comparison. We did not have diet or microbiome data to consider for these dental analyses and suggest that these kinds of data should be always incorporated in the future. Significant results were found only when the covariables mental illness and oral clinical data were added to the analysis.

## Introduction

Smoking is a leading cause of preventable death, resulting in more than 7 million deaths annually worldwide and a huge impact on global health. There are around 1 billion smokers in the world, with 80% living in low and middle-income countries [1]. These smokers represent approximately one-third of the global population aged 15 years or older. Even knowing the serious consequences with which smoking is associated, such as high rates of cancer, cardiovascular and pulmonary diseases, and consequently death, about 45.3 million adults in the U.S. smoke cigarettes, and more than 68% claim to want to quit [2].

After starting smoking, the individual can rapidly develop dependence on tobacco, even in the case of young and irregular smokers [3]. Soon after smoking the first cigarette, the neurophysiological processes underlying tobacco dependence are initiated [4,5]. The younger one starts smoking, the greater the risk of addiction in later stages of life [6]. Tobacco dependence occurs through nicotine, which is the main psychoactive component in tobacco [7].

The data on the effect on tobacco is even more contundent in people with mental illness. A meta-analysis of more than 40 studies conducted in 20 countries showed that smokers with severe mental illness were significantly heavier smokers than smokers in the general population. In addition, the prevalence of smoking in patients with schizophrenia was 62%, which represents 5.3 times more than in the general population [8]. In Western countries, the prevalence of smokers in patients with bipolar disorder is 66%, and 60% of patients with depression also smoke [9]. A national survey of people living with psychotic illness in Australia found that 73% of men and 56% of women smoked tobacco [10].

In general, cigarette smoking addiction is influenced by genetic factors [11]. Heredity for initiation of smoke is estimated to be approximately 48–60% [12, 13, 14], and heritability seems to be higher in men, but the difference between the sexes is small [15].

We still do not know why people with mental illnesses smoke excessively but knowing that the opioid and endogenous dopaminergic systems are involved in the strengthening of smoking, the study of these genes is justified. Hirasawa-Fujita *et al*. [16] observed an association among smoking, schizophrenia and the opioid receptor *OPRM1*, and that an alteration of the *DRD2* (dopamine D2) function increased the smoking behavior in women with schizophrenia. The mu-opioid receptor (MOR), encoded by *OPRM1*, is a natural regulator of analgesic pain response and also controls the gratifying effects of many drugs of abuse such as opioids, tobacco, and alcohol [17]. Vink et al. [18] performed a genome-wide association study comparing ever smokers with never smokers and current smokers versus nonsmokers and found evidence of association between OPRM1 and smoking initiation by network-based analysis.

In the study of infectious diseases, affected and unaffected individuals are typically recruited from endemic areas where groups are naturally exposed to the microbial challenge and the resistant and susceptible phenotypes are thus unveiled [19, 20]. However, in the context of smoking, the possibility to impact exposure by discouraging people to smoke interferes with the exposure factor, and consequently a population of non-smokers (the usual control of traditional case–control smoking studies) is theoretically composed of both susceptible and resistant subjects, although their genotypic nature remains uncertain. Therefore, it is possible to affirm that the traditional case–control smoking studies clearly disregard the classic case–control study definition, which states that a case–control study is designed to determine if an exposure is associated with an outcome [21]. Here we hypothesize that a reappraisal of the case–control design based in the observance of the case–control study definition may significantly impact the odds of identification of genetic factors associated with smoking addiction risk. In this context, we propose that patients that smoke only occasionally fulfill the exposure concept and could be a more suitable control for case–control studies representing an apparent resistant phenotype in opposition to uncertain phenotype of subjects that never smoked. To test our hypothesis, in this study we compared genotype frequencies of markers of *OPRM1* by the traditional case–control approach (i.e. smokers versus non-smokers) and the proposed new design (heavy smokers versus light smokers). In addition, to evaluate a genetic association in different groups of smokers and covariates such as mental illness and clinical dental data.

## Materials and methods

All subjects were participants of the Dental Registry and DNA Repository (DRDR) project at the University of Pittsburgh School of Dental Medicine [University of Pittsburgh Institutional Review Board (IRB) approval # 0606091]. This project was started in September 2006 and, as of this date, all the patients treated at the Dental Clinics of the University are invited to be part of the registry. All subjects provided written informed consent authorizing the extraction of dental and medical information from their records and provided a saliva sample for future genetic studies. All 5,800 subjects recruited by July of 2017 were included in this study. The data extracted from the registry database included clinical information and self-reported medical history.

### Definition of controls from published studies looking at smoking and mental illness

In order to confirm how case-control studies on smokers define their appropriate controls for smokers with mental illness, we performed a systematic review in June 2017 with the question (PECO): "What would be the best controls used for a group of dependent smokers with mental illness?". The search was performed using the keywords: "tobacco smoking" OR "nicotine dependence" AND "control group" AND mental. We obtained a total of 61 articles (12 in Cochrane, 29 in Scopus and 20 in PubMed) and, after reading each abstract, we excluded 56 articles because they did not show the group selection information, were not related only to smokers, or were related to cigarette cessation tests. In the end, five articles (four case-control articles and one cohort) were selected for analysis (Table 1). During the peer-review process, we repeated our search in February 2019 with the quotation markers and without the quotation marks. These searches gave us an additional four papers when the search was done with quotations and 191 papers when no quotations were used. However, no papers could be added to the ones we selected in our original search.

**Table 1:** Identified studies describing controls for smokers with mental illness.

Reference	Model	Checklist	Control Used
**Smolka *et al*. 2004 [22]**	Case-Control	STROBE	37 smokers (more than 20 cigarettes / day) and 18 non-smokers.
**Guney *et al*.** **2009 [23]**	Case-Control	STROBE	32 smokers (more than 15 cigarettes / day for more than 2 months) and 32 controls that never smoked.
**Yip *et al*.** **2009 [24]**	Case-Control	STROBE	10 without mental illness and non-smoker, 9 without mental illness and smoker, 10 with schizophrenia and non-smokers, 32 with schizophrenia and smokers.
**Boumaza, Lebain and Brazo 2015 [25]**	Cohort	STROBE	45 smokers and 27 non-smokers (no smoking quantity / frequency data).
**Zhang *et al*.** **2015 [26]**	Case-Control	STROBE	690 patients with schizophrenia (522 smokers and 168 non-smokers) and 628 controls without schizophrenia (322 smokers and 306 non-smokers). Smokers (more than 1 cigarette / day for more than 1 year) and non-smokers (less than 100 cigarettes in a lifetime)

We observed a lack of concordance among the studies for the definition of "smoker", with no information related to the quantity and frequency of tobacco used. In addition, the definition of "non-smokers" also did not appear standardized. Zhang *et al*. [26] considered nonsmokers those who smoked less than 100 cigarettes throughout their lives, while Guney *et al*. [23] considered non-smokers those who never smoked. Since we could not answer the question of the systematic review and to avoid grouping participants who smoked little or very much in a single group of smokers, and since quantity and frequency of tobacco may be influenced by the genetic predisposition to dependence, and to consider the existence of ex-smokers, we used a few different groups as comparison for the genetic association study we performed.

### Study participants

In order to select the patients who would be part of this study, we classified smokers into different groups, according to the amount and frequency of smoking, and also in two subcategories, with and without mental illness: (1) heavy smokers with mental illness; (2) heavy smokers without mental illness; (3) ex-smokers with mental illness; (4) ex-smokers without mental illness; (5) light smokers with mental illness; (6) light smokers without mental illness; (7) non-smokers with mental illness; and (8) non-smokers without mental illness.

Light smokers were those who smoked less than 10 cigarettes (half a pack) per day and heavy smokers, those who smoked more than 11 cigarettes per day. Ex-smokers were considered those who had stayed at least five months prior to the survey, without smoking. Nonsmokers were those who had never smoked in their lives, according to the report of the medical history. The cutoff point between light and heavy smokers was decided based on the way the data were reported in our project: smoking up to half a pack or more than half a pack of cigarettes per day.

For the covariant mental illness, thinking of not subdividing the groups in relation to all the mental illnesses found in the records and thus obtaining very small and unrepresentative samples from each group, we decided to aggregate all the mental illnesses found: depression, bipolar disorder, anxiety, schizophrenia, panic disorders, high stress, eating disorders, and, most often, the association of more than one psychic disorder. In this way, we divided the patients according to presence or absence of mental illness.

### Dental clinical data

Dental clinical data were collected from the medical records of the study participants: tooth loss, dental caries, and periodontitis. Dental caries experience data were obtained through the DMFT (Decayed, Missing due to caries, Filled Teeth) index and tooth loss was defined when there was a lack of one or more teeth, extracted due to dental caries or periodontitis. Periodontitis was defined in individuals presenting at least three teeth exhibiting sites of clinical attachment loss equal or greater to 5 mm in two different quadrants. All clinical conditions were evaluated by professionals in training under the supervision of experienced dentists. These data were dichotomized as to their presence or absence. Dental caries and periodontitis were also analyzed in combination (individuals with both conditions versus individuals with none of the conditions) per the current joint suggestion of the European Organization for Caries Research and the European Federation of Periodontology (EFP) [27].

### OPRM1

In addition to the dopaminergic, serotonergic and nicotinic receptor genes, there are other genes linked to addictive substances that can be found in the endogenous opioid system. The opioid system is involved in the mesolimbic reward system, which is of great importance in addictive behaviors, such as smoking [28]. Within this system, a gene often studied is *OPRM1* [28, 29, 30, 31]. *OPRM1* occupies a region of 200kb in the long arm of chromosome 6 and is a receptor for endogenous opioids, such as beta-endorphin and endomorphin [17]. We selected two markers flanking *OPRM1* (rs553202 and rs7755635) in 6q24-q25 that were not in strong linkage disequilibrium with each other and had good heterozygosity (30% or more) for this study.

### DNA extraction and genotyping

Genomic DNA was extracted from oral cells of unstimulated saliva (2mL) collected using Oragene DNA kits (DNA Genotek, Ottawa, Ontario, Canada) and extraction protocol according to the manufacturer's specifications.

Genotyping was performed by polymerase chain reactions using the Taqman method [32] with an ABI PRISM QuantStudio 6 Flex instrument (Foster City, CA). The pre-designed probes were supplied by Applied Biosystems (Foster City, CA).

### Statistical analysis

Data were analyzed using the SPSS (Statistical Package for the Social Sciences), version 22 (IBM SPSS Statistics, Chicago, IL) package, and the PLINK software [33].

Linkage disequilibrium between the two genetic markers tested was calculated using D’ statistics implemented in the Haploview 4.2 software [34].

Logistic regression analyses of each genetic marker was performed as implemented in PLINK. Dental clinical data (tooth loss, caries and periodontitis) were used as covariates in logistic regression analysis. The established value for alpha was 5%, and the Hardy-Weinberg equilibrium was evaluated by the chi-square test with a degree of freedom for each marker.

The samples were tested for association with *OPRM1* as follows:

1. Heavy and Light Smokers versus Ex-Smokers and Non-Smokers

2. Heavy smokers versus Light Smokers

3. Heavy Smokers versus Ex-Smokers

4. Heavy smokers versus Non-Smokers

5. Heavy Smokers versus Light Smokers, Ex and Non-Smokers

6. Heavy Smokers versus Light Smokers and Ex-Smokers

7. Heavy Smokers, Light Smokers and Ex-Smokers versus Non-Smokers

8. Light Smokers versus Non-Smokers

9. Light Smokers versus Ex-Smokers

10. Ex-Smokers versus Non-Smokers

Power calculations [35], assuming that our marker alleles were in complete linkage disequilibrium with the genetic variant contributing to smoking dependence, showed that we have a sufficient statistical power of 80% under the following parameters specified in the calculations: high-risk allele frequency set at 0.1, the disease prevalence in the general population set at 0.2, and the genotype risks for the Aa and AA genotypes relative to the baseline aa genotype risk of at least 1.5 and 2.5. If the relative risk for heterozygotes is 1.5, we need at least 700 individuals in each comparison group for 80% power. If the relative risk for heterozygotes is 2.0, 200 individuals in each comparison group will give 80% power.

## Results

Initially, a total of 2,017 patients were included in this study, which were allocated to eight distinct groups: (1) heavy smokers with mental illness (n = 165), (2) heavy smokers without mental illness (n = 450), (3) ex-smokers with mental illness (n = 126), (4) ex-smokers without mental illness (n = 396), (5) light smokers with mental illness (n = 68), (6) light smokers without mental illness (n = 226), (7) non-smokers with mental illness (n = 353), (8) non-smokers without mental illness (n = 233). However, after processing of all saliva in preparation for genotyping, 120 samples were excluded due to poor quality and the final sample consisted of 1,897 patients (1: 152; 2: 447; 3: 110; 4: 372; 5: 64; 6: 217; 7: 325; and 8: 210).

Table 2 describes the studied sample according to sex, age, and geographic origin. Even though the comparison groups were not matched by sex, age, or geographic origin, we did not observe differences in their distribution.

**Table 2:** Samples included in the study (1: heavy smokers with mental illness, 2: heavy smokers without mental illness, 3: ex-smokers with mental illness, 4: ex-smokers without mental illness, 5: light smokers with mental illness, 6: light smokers without mental illness, 7: non-smokers with mental illness, and 8: non-smokers without mental illness).

	1 n = 152	2 n = 447	3 n = 110	4 n = 372	5 n = 64	6 n = 217	7 n = 325	8 n = 210
**Sex** Female	72 (47.37%)	221 (49.44%)	59 (53.63%)	190 (51.07%)	31 (48.44%)	101 (46.54%)	166 (51.08%)	104 (49.52%)
Male	80 (52.63%)	226 (50.56%)	51 (46.37%)	182 (48.93%)	33 (51.56%)	116 (53.46%)	159 (48.92%)	106 (50.48%)
**Age (mean and standard deviation)**	46.11 ±12.80	43.23 ±13.98	51.96 ±14.40	52.04 ±18.16	44.87 ±15.30	40.14 ±15.74	49.99 ±14.98	43.97 ±18.82
**Geographic origin** White	129 (84.87%)	360 (80.54%)	88 (80%)	302 (81.18%)	45 (70.31%)	125 (57.60%)	271 (83.39%)	123 (58.57%)
Black	22 (14.47%)	82 (18.34%)	21 (19.09%)	64 (17.20%)	18 (28.13%)	83 (38.25%)	50 (15.38%)	62 (29.52%)
Asian	01 (0.66%)	05 (1.12%)	01 (0.91%)	06 (1.62%)	01 (1.56%)	09 (4.15%)	04 (1.23%)	25 (11.91%)

The 10 case-control group comparison options evaluated were in Hardy Weinberg equilibrium. D’ between the two markers was 0, suggesting they provide independent information about the locus. There was no significant association between *OPRM1* (rs553202 and rs7755635) and smoking (Table 3).

**Table 3:** Genetic analysis (allelic association and Hardy Weinberg) of gene *OPRM1* and smoke.

Comparison	CHR	SNP(*OPRM1*)	A1	F_A	F_U	A2	p-value	OR	HW
**1.**	6	rs553202	T	0.28	0.27	C	0.67	1.04	0.179
	6	rs7755635	C	0.39	0.40	A	0.29	0.93	0.845
**2.**	6	rs553202	T	0.28	0.29	C	0.61	0.94	1.000
	6	rs7755635	C	0.38	0.41	A	0.21	0.88	0.885
**3.**	6	rs553202	T	0.28	0.30	C	0.45	0.92	0.044
	6	rs7755635	C	0.38	0.40	A	0.32	0.91	0.845
**4.**	6	rs553202	T	0.28	0.26	C	0.35	1.11	0.865
	6	rs7755635	C	0.38	0.41	A	0.12	0.88	0.487
**5.**	6	rs553202	T	0.28	0.28	C	0.95	0.99	0.179
	6	rs7755635	C	0.38	0.41	A	0.10	0.89	0.845
**6.**	6	rs553202	T	0.28	0.29	C	0.44	0.93	0.119
	6	rs7755635	C	0.38	0.40	A	0.19	0.90	0.373
**7.**	6	rs553202	T	0.28	0.26	C	0.12	1.16	0.179
	6	rs7755635	C	0.39	0.41	A	0.29	0.92	0.845
**8.**	6	rs553202	T	0.29	0.26	C	0.20	1.18	0.767
	6	rs7755635	C	0.41	0.41	A	0.96	0.99	0.609
**9.**	6	rs553202	T	0.29	0.29	C	0.90	0.98	0.079
	6	rs7755635	C	0.40	0.40	A	0.70	1.04	0.249
**10.**	6	rs553202	T	0.29	0.26	C	0.10	1.20	0.076
	6	rs7755635	C	0.40	0.41	A	0.60	0.95	0.644

Logistic regression analyses with mental illness as covariate showed associations between heavy smoking and *OPRM1* for two comparisons of different case-control frameworks (Table 4):

4 = Heavy Smokers versus Non-Smokers
7 = Heavy, Light and Ex-Smokers versus Non-Smokers

**Table 4:** Logistic regression analysis results with covariates: Mental illness and Oral Conditions.

Comparison	SNP (*OPRM1*)		OR	p-value	Factor
**1**	rs553202	Tooth loss	1.099	0.48	
		Dental caries	1.101	0.35	
		Periodontitis	0.9599	0.67	
		Dental caries + periodontitis	1.079	0.60	
		Mental illness	1.208	0.08	
	rs7755635	Tooth loss	1.128	0.32	
		Dental caries	0.9986	0.99	
		Periodontitis	0.9866	0.88	
		Dental caries + periodontitis	0.9925	0.95	
		Mental illness	1.184	0.09	
**2**	rs553202	Tooth loss	1.361	0.14	
		Dental caries	0.8058	0.19	
		Periodontitis	0.6759	0.01	Protection
		Dental caries + periodontitis	0.5553	0.009	Protection
		Mental illness	0.9725	0.86	
	rs7755635	Tooth loss	1.23	0.28	
		Dental caries	0.8714	0.36	
		Periodontitis	0.6682	0.004	Protection
		Dental caries + periodontitis	0.5649	0.006	Protection
		Mental illness	1.006	0.97	
**3**	rs553202	Tooth loss	0.9153	0.64	
		Dental caries	1.281	0.07	
		Periodontitis	0.8358	0.16	
		Dental caries + periodontitis	1.008	0.54	
		Mental illness	1.062	0.43	
	rs7755635	Tooth loss	0.9008	0.54	
		Dental caries	1.214	0.13	
		Periodontitis	0.8877	0.32	
		Dental caries + periodontitis	0.9928	0.97	
		Mental illness	1.069	0.61	
**4**	rs553202	Tooth loss	1.48	0.02	Risk
		Dental caries	0.8604	0.27	
		Periodontitis	0.8491	0.20	
		Dental caries + periodontitis	0.7717	0.26	
		Mental illness	1.345	0.04	Risk
	rs7755635	Tooth loss	1.49	0.01	Risk
		Dental caries	0.7923	0.06	
		Periodontitis	0.8376	0.13	
		Dental caries + periodontitis	0.6819	0.02	Protection
		Mental illness	1.315	0.03	Risk
**5**	rs553202	Tooth loss	1.234	0.16	
		Dental caries	0.9905	0.93	
		Periodontitis	0.8082	0.04	Protection
		Dental caries + periodontitis	0.7897	0.34	
		Mental illness	1.14	0.25	
	rs7755635	Tooth loss	1.203	0.17	
		Dental caries	0.9527	0.63	
		Periodontitis	0.8222	0.04	Protection
		Dental caries + periodontitis	0.7653	0.06	
		Mental illness	1.146	0.20	
**6**	rs553202	Tooth loss	1.062	0.71	
		Dental caries	1.086	0.50	
		Periodontitis	0.7771	0.03	Protection
		Dental caries + periodontitis	0.8036	0.21	
		Mental illness	1.028	0.82	
	rs7755635	Tooth loss	1.011	0.94	
		Dental caries	1.082	0.48	
		Periodontitis	0.8045	0.04	Protection
		Dental caries + periodontitis	0.8185	0.20	
		Mental illness	1.045	0.71	
**7**	rs553202	Tooth loss	1.432	0.01	Risk
		Dental caries	0.8163	0.08	
		Periodontitis	0.9926	0.95	
		Dental caries + periodontitis	0.8821	0.42	
		Mental illness	1.319	0.02	Risk
	rs7755635	Tooth loss	1.469	0.003	Risk
		Dental caries	0.7556	0.008	Protection
		Periodontitis	0.9609	0.69	
		Dental caries + periodontitis	0.7739	0.07	
		Mental illness	1.287	0.02	Risk
**8**	rs553202	Tooth loss	1.11	0.61	
		Dental caries	1.006	0.97	
		Periodontitis	1.278	0.12	
		Dental caries + periodontitis	1.376	0.15	
		Mental illness	1.375	0.21	
	rs7755635	Tooth loss	1.199	0.33	
		Dental caries	0.8789	0.39	
		Periodontitis	1.271	0.10	
		Dental caries + periodontitis	1.175	0.44	
		Mental illness	1.304	0.09	
**9**	rs553202	Tooth loss	0.7119	0.12	
		Caries	1.515	0.01	Risk
		Periodontitis	1.183	0.29	
		Dental caries + periodontitis	1.795	0.01	Risk
		Mental illness	1.094	0.60	
	rs7755635	Tooth loss	0.7611	0.18	
		Dental caries	1.324	0.07	
		Periodontitis	1.295	0.08	
		Dental caries + periodontitis	1.704	0.01	Risk
		Mental illness	1.05	0.76	
**10**	rs553202	Tooth loss	1.555	0.02	Risk
		Dental caries	0.683	0.008	Protection
		Periodontitis	1.054	0.70	
		Dental caries + periodontitis	0.7731	0.18	
		Mental illness	1.26	0.12	
	rs7755635	Tooth loss	1.581	0.006	Risk
		Dental caries	0.6657	0.002	Protection
		Periodontitis	0.9735	0.83	
		Dental caries + periodontitis	0.6866	0.03	Protection
		Mental illness	1.243	0.11	

1. Heavy Smokers and Light Smokers versus Ex-Smokers and Non-Smokers, 2. Heavy Smokers versus Light Smokers, 3. Heavy Smokers versus Ex-Smokers, 4. Heavy Smokers versus Non-Smokers, 5. Heavy Smokers versus Light Smokers, Ex-Smokers and Non-Smokers, 6. Heavy Smokers versus Light Smokers and Ex-Smokers, 7. Heavy Smokers, Light Smokers and Ex-Smokers versus Non-Smokers, 8. Light Smokers versus Non-Smokers, 9. Light Smokers versus Ex-Smokers, 10. Ex-Smokers versus Non-Smokers

Our results showed that this association is only present when we compared the group of heavy smokers (the heavy smoking group alone or associated with all individuals who smoke and have smoked) with the group of those who have never smoked in their lives (non-smokers).

Logistic regression, using dental clinical data as independent covariables and correlating them with *OPRM1* genotypes and tobacco dependence, found some associations. Tooth loss in the presence of *OPRM1* genetic variation was more likely in people who currently smoked or smoked in the past (comparison 4—Heavy smokers versus Non-Smokers, 7—Heavy Smokers, Light Smokers and Ex-Smokers versus Non-Smokers, and 10—Ex-Smokers versus Non-Smokers). These data show that the chance for tooth loss is higher for smokers, regardless of the amount and frequency of tobacco use, compared to individuals who never smoked. Dental caries in the presence of *OPRM1* genetic variation was less likely to occur for comparisons 7—Heavy Smokers, Light Smokers and Ex-Smokers versus Non-Smokers and 10—Ex-Smokers versus Non-Smokers, and more likely for comparison 9—Light Smokers versus Ex-Smokers. This result suggests dental caries in less likely in ex-smokers when compared to those who never smoked. However, when active smokers were considered, dental caries was more likely to occur compared to those who stopped smoking. Periodontitis in the presence of *OPRM1* genetic variation was less likely to occur in smokers in comparisons 2—Heavy smokers versus Light Smokers, 5—Heavy Smokers versus Light Smokers, Ex and Non-Smokers, and 6—Heavy Smokers versus Light Smokers and Ex-Smokers, suggesting that heavy smokers were less likely to have periodontitis, when compared to light smokers. When we analyzed individuals with both dental caries and periodontitis in comparison with those without any of these two conditions, we observed that individuals who smoked more were less likely to have dental caries and periodontitis. Similarly, the comparison done with individuals who never smoked or were light smokers (2—Heavy smokers versus Light Smokers, 4—Heavy smokers versus Non—Smokers, 10—Ex—Smokers versus Non—Smokers) show the same results. However, when we evaluated group 9—Light Smokers versus Ex-Smokers, we observed that there was an increased risk of having dental caries associated with periodontitis for those who smoked compared to those who stopped smoking (Table 4).

## Discussion

It should be emphasized the importance of the development of a research in this field because of the high percentage of smokers in the world [1], particularly among individuals with mental illness [8], linked to the possibility of the dependence to nicotine is correlated with genetic factors.

We did not find an association between smoking and *OPRM1*, which corroborates with the results of Kleinjan *et al*. [31]. On the other hand, other studies described significant association between smoking and *OPRM1*. *OPRM1* was shown to be associated with higher reward activity in response to nicotine intake because of the greater effectiveness at the μ-opioid receptor to β-endorphin binding, which leads to increased dopaminergic activity. This potential for opioid receptor binding appeared to be significantly higher for the A allele of *OPRM1* (A118G/rs1799971) [30]. A meta-analysis performed by Arias *et al*. [28] on the A118G variant of the *OPRM1* gene in relation to other substances (alcohol, opioids and heroin) evaluated 19 case-control studies and showed that the *OPRM1* gene did not affect the risk of dependence on these substances. However, of the 7 studies that had a significant effect on the A118G variant, 4 of them reported that the G allele was the risk variant, while the other 3 studies demonstrated that G variant was protective for dependence. Another meta-analysis performed by Schwantes-An *et al*. [36] investigated the non-specific risk of *OPRM1* with dependence on "general" substances (alcohol, opioids, marijuana, cocaine and nicotine), comparing substance-dependent cases with controls that were not dependent on the substances evaluated. The G allele of rs1799971 has been shown to have a modest protective effect on the risk of general substance dependence in samples of European ancestry. Similar effects have been reported for each individual substance, probably due to the small sample size. The authors concluded that rs1799971 contributed to the mechanisms of dependency liability that are shared between different substances that cause addiction. The divergence of the results among the studies may be related to several factors, such as the use of different genetic markers (rs1799971, rs553202 and rs7755635), sample size, as well as different compositions of the case / control groups.

Based on studies that we found on our systematic review, we observed a lack of concordance among the studies for the definition of "smoker", with no information related to the quantity and frequency of tobacco use in these studies. In addition, the definition of "non-smokers" also did not appear standardized in our systematic review. Therefore, we divided the group of smokers into four categories (heavy smoker, light smoker, ex-smoker and non-smoker). The characterization of smokers that we used, with four different groups in the same study, was not observed in the literature and makes our study unique. For example, Verde *et al*. [37] reported that smokers were those that smoked more tan 10 cigarettes per day at the time of the study and other studies that analyzed the association between smoking and genetic variants did not mention data on the quantity and frequency of tobacco use among smokers [38, 39]. We compared the addiction to smoke risk odds using the traditional approach (i.e. smokers versus non-smokers) and our proposed new design (heavy smokers versus light smokers).

Nicotine is the essential factor in tobacco addiction, and its action on nicotinic cholinergic receptors in the brain is primarily to increase the release of various neurotransmitters, including dopamine, noradrenaline, acetylcholine, glutamate, GABA, vasopressin, endorphin and others. The release of neurotransmitters is thought to mediate psychological effects such as arousal, relaxation, cognitive improvement, stress relief, and depression. Repeated exposure to nicotine increases its locomotor and booster effects in rodents, a phenomenon known as sensitization, common to psychostimulant drugs. In addition, repeated administration of nicotine enhances the dopamine stimulatory effects [40]. High doses of nicotine produce reward stimuli greater than low doses, and rapid arrival to the brain brings more reward than slow arrival. It is determinant for the establishment of dependence that the cigarette offers high concentrations of nicotine rapidly to the central nervous system [41]. The μ-opioid receptor (*OPRM1*) plays an important role in nicotine dependence because of its ability to bind β-endorphins and enkephalins, which are released after nicotine ingestion. This, in turn, leads to the release of dopamine into various areas of the brain, providing feelings of reward by increasing the additive properties of the cigarette [31]. Thus, the importance of smoking classification according to tobacco quantity and frequency is highlighted. Even with the classification of the groups and the various combination options for comparisons, we did not find any significant results associating smoking and the *OPRM1* gene, which suggests indeed *OPRM1* influence in smoking addiction may be too small to ever become of clinical significance or target for therapy, unless underlying mental disorder is present. Indeed, we found significant results for the association between smoking in individuals with mental illness and *OPRM1*. Our results showed that this association is only present when we compared the group of heavy smokers (the heavy smoking group alone or associated with all individuals who smoke and have smoked) with the group of those who have never smoked in their lives (non-smokers). Zerdazi *et al*. [42] also showed a similar result, finding an association between *TLR4* and bipolar disorder. Hirasawa-Fujita *et al*. [16] observed that *OPRM1* is associated with increased smoking in patients with schizophrenia, and *DRD2* has also been associated with increased smoking behavior in women with schizophrenia. Thus, according to our results and the above-mentioned reports, we showed that genetic variation in *OPRM1* associates with smoking addiction in patients with mental illness.

Oral health in indeed impacted by tobacco dependence. Tooth loss is higher for smokers, regardless of quantity, when compared to individuals who never smoked (non-smokers). These data corroborate the results of a cohort study that assessed the effects of smoking on tooth loss even after cessation of smoking, with a 46-year follow-up in northern Finland, that showed that smoking has effects on the loss of teeth in the long run [43]. The results for dental caries were less clear: it was less likely for the ex-smokers when compared to those who never smoked, and more likely for smokers compared to those who quit smoking. The last result represents that the cessation of smoking can lead to improvement in oral health and, consequently, decrease of the caries experience. Benedetti *et al*. [44], in a systematic review of tobacco and dental caries, showed that smoking was associated with an increased risk of dental caries. However, the poor overall quality of the studies did not produce validation for such association. Badel *et al*. [45] investigated the experience of caries and tobacco use in 19-year-old Croats and concluded that non-smokers had fewer decayed teeth. In our study, periodontitis was less likely in heavy smokers compared to light smokers. The high number of cigarettes would not bring more harm to the periodontium compared to daily smokers with low cigarettes (less than 10 cigarettes / day). This result is controversial in the literature; some studies show consistent data for tobacco as a risk factor for periodontal disease [46, 47, 48, 49] and other studies report that there is no association, but that this non-association may be weak or take more time to happen [50, 51]. Smoking is identified as a risk factor for periodontitis, but risk estimates diverge between studies [47].

Combining dental caries and periodontitis as a composite phenotype has been suggested [27]. Since smoking impacts both conditions, as well as increases the chance for their ultimate negative outcomes (tooth loss) to occur, the suggestion is warranted. Our results consistently showed that the individuals used for comparison (light smokers, ex-smokers, non-smokers, or a combination of them) tended to have more often dental caries and periodontitis in the presence of *OPRM1* genetic variation. We considered the interaction between mental illness and oral health outcomes. This interest has occurred because this interaction has been studied. We also observed that tooth loss, caries, and periodontitis are "outcomes" present in both mental illness and tobacco dependence. Therefore, it is critical to emphasize that poor general health can lead to poor oral health. Although much emphasized lately [52], our data do not support the idea that poor oral health is driving the risk for mental illness.

Being concerned about multiple testing, we avoided applying the strict Bonferroni correction and increasing the type II error. If we had used Bonferroni correction, we would have lowered the alpha to 0.0025 (0.05/20). Therefore, here we report all results with P values below 0.05. However, our data must be carefully interpreted because it is expected that some of the P values below 0.05 can be due to chance. Also, our power analysis showed that we had sufficient statistical for a range of comparisons. But we know that we could have a lack of association that may be due to lack of statistical power.

The reason why non-smokers or light smokers were more likely to have dental caries associated with periodontitis when compared to heavy smokers is intriguing. One possibility is that the population studied is possibly exposed in high numbers to second hand smoking, low quality air, or other chemical irritants and that would account for the non-intuitive results. These subjects are from the greater Pittsburgh area; for the most part, individuals with low socioeconomic status and hence high risk for all oral and overall health problems tend to be over-represented in the patient pool [53].

## Conclusions

In this study, no associations were found between *OPRM1* and smoking, but when added to the analysis, mental illness and oral clinical data, significant associations were found. Our data show that the comparison group matters depending on the oral health outcome being measured. Tooth loss risk increases in the presence of *OPRM1* genetic variation if one smokes at any frequency. Impact of *OPRM1* in dental caries or periodontitis risk when someone smokes appears to depend of other factors that were not measured. The counterintuitive results for dental caries and periodontitis in these analyses may suggest that the effect of smoking in these diseases may be the consequence of dietary or microbiological changes that happen when someone stops smoking. We did not have diet or microbiome data to consider for these analyses and suggest that these kinds of data should be always incorporated in the future. Another limitation of this study is that the data collection was performed by different students / teachers, as it was carried out for several years. Because we work with secondary data from this database, we cannot prevent this. We also suggest that multiple comparison groups comprised by individuals that never smoked and individuals that smoked at one point and stopped should be utilized.

## Supporting information

S1 File Raw clinical data. Raw clinical data.
(XLSX)
